# Analysis of the multicomponent ALEX array data to examine patterns of sensitization in Cape Town, South Africa

**DOI:** 10.3389/falgy.2025.1572509

**Published:** 2025-05-07

**Authors:** Sarah Pedretti, Alexander Sittmann, Arné Von Hagen, Jonny Peter

**Affiliations:** ^1^ Allergy and Immunology Unit, University of Cape Town Lung Institute, Cape Town, South Africa; ^2^ University of Cape Town, Cape Town, South Africa; ^3^ Department of Medicine, Division of Allergy and Clinical Immunology, University of Cape Town, Cape Town, South Africa

**Keywords:** allergy, major allergens, sensitization, ALEX, cross-reactivity, Cape Town

## Abstract

**Introduction:**

This study analysed allergen sensitization patterns in Cape Town, a biodiversity-rich region with a Mediterranean climate, using ALEX® and ALEX²® multiplex component-resolved diagnostics tools. It aimed to address gaps in allergen sensitisation pattern data and complement aerobiological monitoring.

**Methods and results:**

A retrospective review of 708 adults and children attending two tertiary allergy clinics (2019–2024) found that house dust mites were the most common allergens, affecting 50%–60% of participants, with Der p 23 particularly prevalent (53%). Grass pollen sensitization was also high (46%), with 85% sensitised to the C4 grass Bermuda. Tree pollen sensitisation occurred in 29% with 14% sensitised to a diverse range of trees but neither London plane nor Cypress currently recommended in limited testing panels. Common food allergens included fruits (30%), seafood (27%), and nuts (25%), often linked to pollen cross-reactivity.

**Conclusion:**

Our study confirms a known pattern of aeroallergen sensitisation for a coastal temperate region, with increasing pollen sensitisation, particular C4 grasses. Clinicians should be aware of the diversity of tree pollen sensitisation, cross-reactivity patterns between food and pollen sensitisations and rates of minor allergen sensitisations for Blomia and animal danders when considering allergen-immunotherapies.

## 1 Introduction

The understanding of local and regional aeroallergen exposure and sensitisation patterns is important for good clinical allergy practice. Anthropogenic impacts on the environment are rapidly changing exposures, particular aerospora exposures such as pollens and fungal spores (1, 2). Therefore, it is becoming ever more important to map and monitor both aerobiology in populated areas, as well as understand local sensitisation patterns, and how they are changing. South Africa (SA) has eight distinct biomes and one of the most biodiverse countries in the world (3). SA also has a considerable burden of allergic diseases, including high rates of severe asthma (1–6); pollen and fungal spores are important drivers (7). The SA pollen monitoring network has been working to expand aerobiological monitoring across the different biomes of SA and most densely populated cities; recently publishing data on seven cities (2). However, there is a major paucity of sensitisation data that can be matched with this aerobiological data to understand the relative importance of different indoor and outdoor allergens, and in particular for different tree and weed species, help to understand which species more likely drive local allergic disease patterns and should be included into testing panels (2). Our study aimed to address this important gap for Cape Town, a city with a Mediterranean climate, in the Western Cape region of SA.

Component-resolved diagnostics (CRD) involves the use of purified or recombinant allergen components to characterise specific molecules that induce sensitisation in atopic individuals (3, 8). CRD has been found to be advantageous, in certain instances, over traditional methods of allergy testing using of whole-allergen extracts—such as skin prick testing (SPT) (4, 9–12). In particular, CRD is useful to differentiate between primary co-sensitisation and cross-reactivity, inform allergen immunotherapy (AIT) product selection, and better understand minor allergen importance (8, 11, 13). CRD has been a relatively recent addition to allergology in developing countries like SA.

The aim of this study was to analyse patterns of sensitisation prevalence among Capetonian individuals undergoing CRD at a tertiary allergy clinic.

## 2 Materials and methods

Our study design was retrospective observational. We considered CRD data from 708 individuals from the Cape Town area, including patients attending two tertiary allergy clinics at either Groote Schuur Hospital Allergy Clinic (state-sector) and the Allergy and Immunology Unit of the University of Cape Town (UCT) Lung Institute (private sector) who underwent allergy testing as part of their diagnostic workup. Testing was at the request of the treating allergist following presentation with diverse set of allergic diseases between January 2019 and July 2024. History of allergic reaction involves any atopic disease including (but not limited to) allergic rhinoconjunctivitis, asthma, atopic dermatitis, anaphylaxis, urticaria, and angioedema thought to be secondary to an exogenous allergen. There were no exclusion criteria for our patients.

Allergen data were collected with the Multicomponent Allergy Explorer (ALEX) array from Micro Array Diagnostics, a CRD tool that quantifies specific immunoglobulin E (sIgE) for a panel of about 300 allergens (14). 100 µl of serum was used for each patient. Two versions of ALEX, ALEX® (282 allergens; panel available on demand) and ALEX²® [300 allergens (15)], differing in their allergy panels, were used across the selected population. Among the 708 patients tested, 554 were tested using ALEX^2^®, and the other 154 with ALEX® from October 2020. Only basic demographics including age were available. This study was approved by UCT Faculty of Health Sciences Human Research Ethics Committee (HREC 368/2024).

Our statistical analysis included simple descriptive statistics. sIgE (per allergen for a patient) was reported in IgE response units (kUA/L, or kilounits of allergen-sIgE per litre); and we defined a patient “sensitised” to an allergen if sIgE ≥0.30 kUA/L. The full 354-allergen complement found across both ALEX versions were used for the allergen-and-source-specific analyses, but we excluded 110 allergens from some of the prevalence-descriptive analyses, as these were found on either ALEX® or ALEX²®, but not on both. We considered an individual sensitized to an allergen group if sensitized to at least one allergen in this group. Details of the allergens in each group is described in Supplementary Table 2.

## 3 Results

### 3.1 Dominant sensitisation patterns

**Figure 1:**
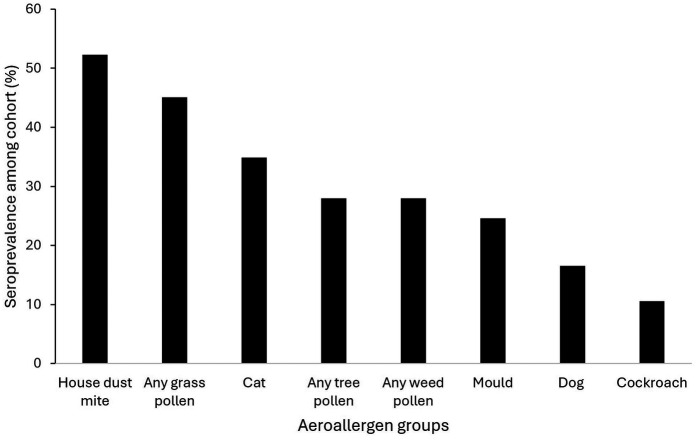
Dominant aeroallergen groups by % sensitised to any allergen in that group (*n* = 708).

Among the 708 patients tested, 154 were tested using ALEX® (146 > 12 years old and 4 < 12 years old, missing age for 4 participants), and the other 554 with ALEX²® (514 > 12 years old and 39 < 12 years old, missing age for 1 participant). Table 1 shows the sensitization (number and prevalence) over the two versions of ALEX by allergen groups in all individuals and stratified by age. In all individuals house dust mites (HDM) was the dominant allergens group with 60.1% and 50.5% of individuals sensitized using ALEX® and ALEX²® respectively, followed by the grass pollen group with 45.8% and 46.6% and pet danders with 35.9% and 40.1%. Figure 1 shows this seroprevalence in descending order including other aeroallergens: HDM, any grass pollen, cat, any tree pollen, any weed pollen, mould, dog and cockroach. Food allergen sensitisation was fourth commonest overall with fruits (30.1%), seafood (26.8%) and nuts (25.5%) using ALEX® and legumes (33.9%), nuts (22.6%) and fruits (20.8%) using ALEX²® as the commonest individual allergens. Results were similar when only considering adults (>12 years old) regardless of the ALEX version used. In the Hymenoptera venoms category honey-bee venom sensitization was present in 11.8% and 22.7% of individuals using ALEX® and ALEX²® respectively. There was no notable difference between the sensitisation patterns of adults and children.

**Table 1:** Sensitisation prevalence by allergen group (*n* = 708).

Overarching category	Allergen group	Total number of patients sensitised to at least one allergen in ALL group	Total number of patients sensitised to at least one allergen in >12 group	Total number of patients sensitised to at least one allergen in <12 group	Total percentage prevalence in ALL groups (%)	Total percentage prevalence (%) in >12 group	Total percentage prevalence (%) in <12 group
Mites and cockroaches	House dust mites	92; 280	86; 258	4; 22	60.1; 50.5	58.9; 50.2	100.0; 56.4
	Cockroaches	29; 67	27; 61	2; 6	19.0; 12.1	18.5; 11.9	50.0; 15.4
	Storage mites	36; 159	33; 343	2; 16	23.5; 28.7	22.6; 27.8	50.0; 41.0
Pollens	Tree pollens	48; 18	47; 171	0; 15	31.4; 33.6	32.2; 33.3	0.0; 38.5
	Grass pollens	70; 258	68; 236	0; 22	45.8; 46.6	46.6; 45.9	0.0; 56.4
	Weed pollens	45; 167	41; 151	2; 16	29.4; 30.1	28.1; 29.4	50.0; 41.0
Dander and epithelia	Pets	55; 202	51; 206	2; 16	35.9; 40.1	34.9; 40.1	50.0; 41.0
	Farm animals	33; 62	30; 55	2; 7	21.6; 11.2	20.5; 10.7	50.0; 17.9
Moulds and yeasts	Moulds	41; 136	40; 128	0; 10	26.8; 24.5	27.4; 24.5	0.0; 25.6
	Yeasts	13; 47	13; 41	0; 6	8.5; 8.5	8.9; 8.0	0.0; 15.4
Foods	Meats	31; 105	28; 96	2; 9	20.3; 19.0	19.2; 18.7	50.0; 23.1
	Fruits	46; 115	44; 106	1; 9	30.1; 20.8	30.1; 20.6	25.0; 23.1
	Vegetables and mushrooms	36; 53	31; 49	3; 4	23.5; 9.6	21.2; 9.5	75.0; 10.3
	Legumes	18; 188	17; 172	1; 16	11.8; 33.9	11.6; 33.5	25.0; 41.0
	Seafood	41; 110	39; 101	1; 9	26.8; 19.9	26.7; 19.6	25.0; 23.1
	Nuts	39; 125	37; 112	1; 13	25.5; 22.6	25.3; 21.8	25.0; 33.3
	Cereals	19; 80	18; 72	1; 8	12.4; 14.4	12.3; 14.0	25.0; 20.5
	Milk	12; 30	11; 25	1; 5	7.8; 5.4	7.5; 4.9	25.0; 12.8
	Spices	8; 50	8; 43	0; 7	5.2; 9.0	5.5; 8.4	0.0; 17.9
	Seeds	9; 64	8; 56	1; 8	5.9; 11.6	5.5; 10.9	25.0; 20.5
	Egg	23; 27	22; 21	1; 6	15.0; 4.9	15.1; 4.1	25.0; 15.4
Hymenoptera venoms	Honey-bee venoms	18; 126	16; 116	1; 10	11.8; 22.7	11.0; 22.6	25.0; 25.6
	Wasp venoms	10; 46	10; 42	0; 4	6.5; 8.3	6.8; 8.2	0.0; 10.3
	Fire ant poison	20^a^	18^a^	2^a^	3.6^a^	3.5^a^	5.1^a^
Other	Ficus	2; 8	2; 6	0; 2	1.3; 1.4	1.4; 1.2	0.0; 5.1
	Latex	15; 45	14; 39	0; 6	9.8; 8.1	9.6; 7.6	0.0; 15.4
	CCD (cross-reactive carbohydrate determinants)	8; 25	8; 21	0; 4	5.2; 4.5	5.5; 4.1	0.0; 10.3
	Parasite	19^a^	17^a^	2^a^	3.4^a^	3.3^a^	5.1^a^

First number represents the tests done with ALEX® and second number the tests done with ALEX^2^®.

^a^ Only in ALEX^2^®.

Supplementary Figure 1 shows the 30 most prevalent individual allergen components. The HDM antigen Der p was the allergen to which the greatest proportion of people tested were sensitised (53.3%), followed by another HDM allergen, Der p 23 (41.8%) and a grass pollen allergen, Phl p 1 (37.0%). Among the 50 most common sensitising allergens, 12 were grass pollen allergens, 11 were HDM allergens, and 9 were pet allergens. Supplementary Table 1 considers well-known molecular allergen groupings showing cross-reactivity within this cohort.

### 3.2 Sensitization patterns to guide panel testing recommendations and immunotherapy

In 2023 the South African Allergic Rhinitis Diagnostic Working Group (ARDWG) recommended a panel of sIgEs (or SPT) to aeroallergens (16). This included: i) two grass pollens—Bermuda and Rye, two tree pollens—Cypress and Plane, HDM *Dermatophagoides pteronissinus* (Der p) and *Blomia (B.) tropicalis*, moulds *Alternaria (A.) alternata* and *Aspergillus (A.) fumigatus*, cat and dog.

Table 2 shows (A) participants sensitised to individual and combinations of target allergens, as well as (B) participants only sensitised to minor allergens. In our cohort of adult (>12 years old) individuals, 8.0% were sensitized to Timothy grass pollen (Phl p, Phl p 1, Phl p 2, Phl p 5.0101, Phl p 6, Phl p 7, Phl p 12) only, while none were sensitized to either Rye (Lol p 1) or Bermuda grass pollen (Cyn d, Cyn d 1) only. Importantly, cross-reactivity to grass pollens were common including: (i) 35.8% of our cohort was sensitized to both Rye and Timothy grasses pollens, (ii) 29.1% to both Bermuda and Timothy grasses pollens, and (iii) 27.8% to both Bermuda and Rye grasses pollens. Only 8.0% was sensitized to both Rye and Timothy grasses pollens but not Bermuda grass pollen and 27.8% of our cohort was sensitized to these 3 grasses pollens together.

Overall, 29.3% of individuals were sensitized to any tree pollen, with 14.4% sensitized to any tree pollen but not Cypress or London plane trees pollens. Considering panel suggested Cypress and Plane tree pollens, only 5.6% were sensitized to London plane tree pollen (Pla a, Pla a 1, Pla a 2, Pla a 3) only and none to Cypress tree pollen (Cup a 1, Cup s) only, while 4.1% of our cohort were sensitized to both Cypress and London plane trees pollens (Table 2A).

**Table 2A:** Sensitization pattern relevant to panel testing and immunotherapy in South Africa (*n* = 659).

Grass pollens	Patients sensitized to specific allergen(s)	Patients sensitized to specific allergen(s) (% of total patients)
Timothy only	53	8.0
Bermuda only	0	0.0
Rye only	0	0.0
Rye and Timothy	236	35.8
Bermuda and Timothy	192	29.1
Bermuda and Rye	183	27.8
Rye and Timothy not bermuda	53	8.0
Rye and Timothy and bermuda	183	27.8
TREE pollens		
London plane tree only	37	5.6
Cypress only	0	0.0
Cypress and London plane tree	27	4.1
Any tree	193	29.3
Any tree not Cypress not London plane tree	95	14.4
House dust mites + storage mites		
European house dust mite only	0	0.0
American house dust mite only	0	0.0
Blomia tropicalis only	0	0.0
American house dust mite and European house dust mite	264	40.1
European house dust mite and Blomia tropicalis	72	10.9
American house dust mite and Blomia tropicalis	58	8.8
American house dust mite and European house dust mite and Blomia tropicalis	58	8.8
Storage mite only	0	0.0
Storage mite not American house dust mite	10	1.5
Storage mite not European house dust mite	2	0.3
Storage mite not American house dust mite not European house dust mite	2	0.3
Moulds		
Alternaria alternata only	0	0.0
Aspergillus fumigatus only	0	0.0
Alternaria alternata and aspergillus fumigatus	22	3.3
Any mould	161	24.4
Any mould not alternaria alternata Not aspergillus fumigatus	4	0.6

Regarding HDM sensitisation patterns, nobody in our cohort was sensitized to European HDM (Der p, Der p 1, Der p 2, Der p 5, Der p 7, Der p 10, Der p 11, Der p 20, Der p 21, Der p 23), American HDM (Der f, Der f 1, Der f 2) or *B. tropicalis* (Blo t, Blo t 5, Blo t 10, Blo t 21) only. Cross-sensitisation between European and American HDMs were common at 40.1%, while only 10.9% and 8.8% had dual sensitizations to *B. tropicalis* and European and American HDM respectively. There were no patients sensitized to storage mites (Aca s, Gly d, Gly d 2, Lep d, Lep d 2, Tyr p, Tyr p 2) alone, with very low rates of 1.5% and 0.3% with sensitization to any storage mite while not reacting to American or European HDM respectively (Table 2A). Table 2B shows that 41.7% of patients with sensitisation to *B. tropicalis*, only demonstrate sensitisation to a minor allergen (Blo t 10, Blo t 21).

**Table 2B:** Major and minor allergens of commonest aeroallergens showing percentage sensitised to only a minor allergen of that source, where applicable.

Allergen group	Allergen source	Major allergen(s) and % prevalence among those sensitised to source	Minor allergen(s)	% Sensitised to any minor allergen and *no* major allergen(s) among those sensitised to source
House dust mites	Blomia tropicalis	Blo t 5 (57.1)	Blo t, Blo t 10, Blo t 21	41.7
	American house dust mite	Der f 1 (76.6), Der f 2 (85.5)	Der f	0.3
	European house dust mite	Der p 1 (64.7), Der p 2 (69.4), Der p 23 (80.2)	Der p, Der p 10, Der p 11, Der p 20, Der p 21, Der p 5, Der p 7	3.3
Moulds	Alternaria alternata	Alt a 1 (98.6)	Alt a, Alt a 6	1.4
	Aspergillus fumigatus^a^	N/A	Asp f, Asp f 1, Asp f 3, Asp f 4, Asp f 6	N/A
	Cladosporium herbarum^b^	Cla h (66.7), Cla h 8 (71.4)	N/A	N/A
	Penicilium chrysogenum^c^	Pen ch (100)	N/A	N/A
Pets	Dog	Can f 1 (52.6)	Can f, Can f 2, Can f 3, Can f 4, Can f 6, Can f_Fd1, Can f male urine	47.4
	Cat	Fel d 1 (89.1)	Fel d, Fel d 2, Fel d 4, Fel d 7	10.9
Grass pollens	Timothy grass	Phl p 1 (84.8), Phl p 5.0101 (50.8)	Phl p, Phl p 12, Phl p 2, Phl p 6, Phl p 7	6.8
	Bermuda grass^b^	Cyn d (83.6), Cyn d 1 (86.7)	N/A	N/A
	Perennial ryegrass^c^	Lol p 1 (100)	N/A	N/A
	Bahia grass^c^	Pas n (100)	N/A	N/A
	Common reed^c^	Phr c (100)	N/A	N/A
	Cultivated rye^c^	Sec c_pollen (100)	N/A	N/A
	Johnson grass^c^	Sor h (100)	N/A	N/A
	Maize pollen^c^	Zea m pollen (100)	N/A	N/A

^a^ No major allergen (≥50% sensitisation among all those sensitised to source).

^b^ No minor allergen (<50% sensitisation among all those sensitised to source).

^c^ Only one allergen on panel.

Considering mould sensitisation patterns, no participants were sensitized to *A. alternata* (Alt a, Alt a 1, Alt a 6) only or *A. fumigatus* (Asp f, Asp f 1, Asp f 3, Asp f 4, Asp f 6) only. Overall, 24.4% of individuals were sensitized to any mould, with 0.6% being sensitized to any mould other than *A. alternata* or *A. fumigatus* (Table 2A)*.*

High rates of minor allergen only sensitisation was notable for dog and cat allergens, with 47.4% and 10.9% of patients sensitised to only dog and cat minor allergens respectively (Table 2B).

### 3.3 Food allergens sensitisation patterns and exploration of pollen-food syndromes

**Figure 2:**
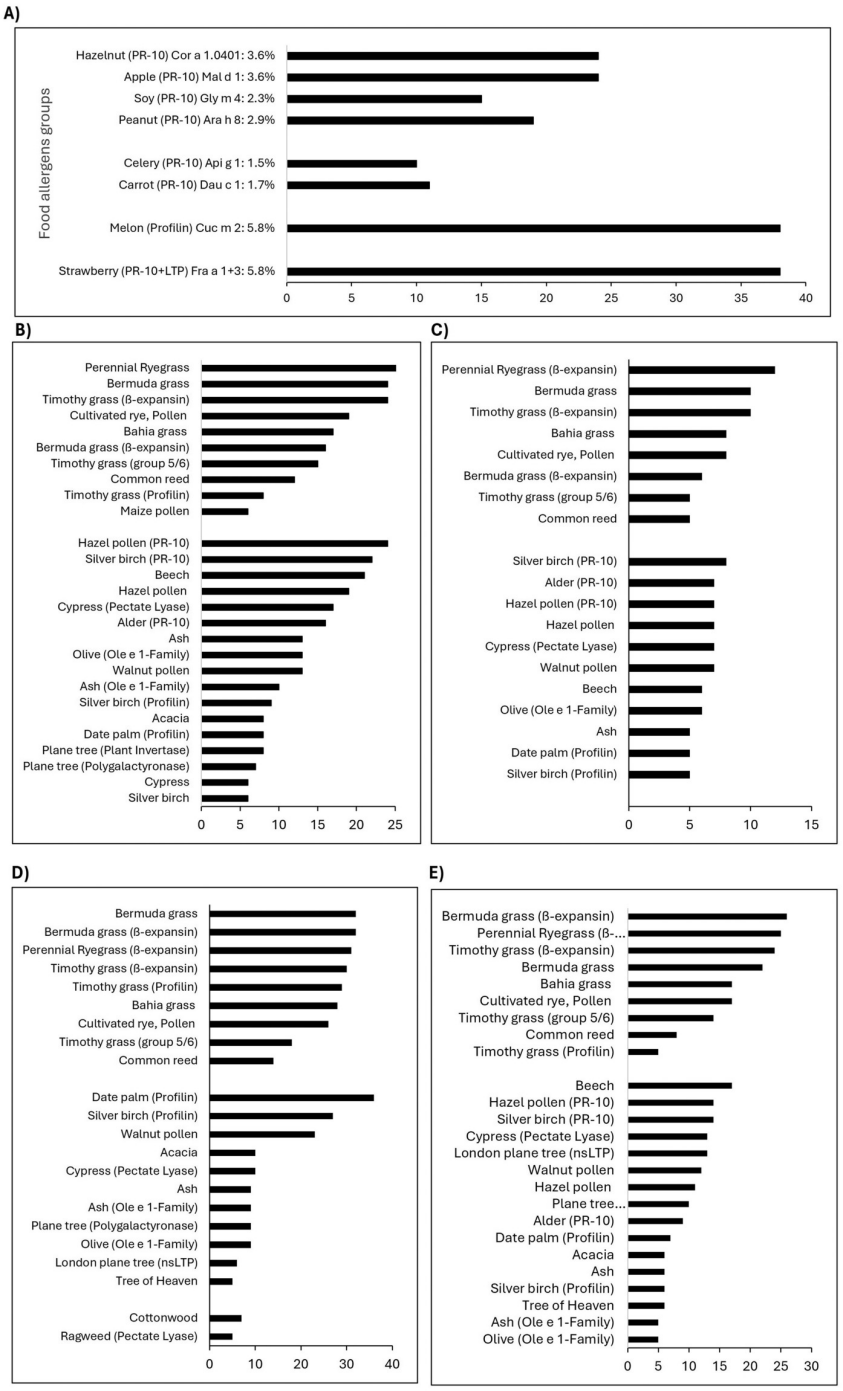
**(A)** Number of adult patients and percentage of the cohort sensitized to one or more PR-10 or profilin proteins in food allergens (*n* = 659). Grass, tree and weed pollens patterns in adult patients sensitized to PR-10 or Profilin proteins in **(B)** hazelnut, apple, soy, peanut (*n* = 43), **(C)** celery, carrot (*n* = 14), **(D)** melon (*n* = 38) and **(E)** strawberry (*n* = 38).

Table 3 shows the common food allergen component sensitisation patterns, from highest to lowest. Figures 2A–E profile pollen sensitisation patterns amongst patients with different PR-10 and profilin sensitisations, which occurred in 14.3% of patients. No clinical food allergy data was available for this cohort, and thus presented data represents only sensitisation without confirmation of clinical hypersensitivity. White bean (Pha v) was the commonest individual sensitisation, with high rates of concomitant sensitization to pollens, including: Timothy (34.0%), Rye (31.9%) and Bermuda grasses (27.1%) (Supplementary Figure 2A); Cypress (16.7%), Walnut (8.3%) and Date palm (7.6%) trees (Supplementary Figure 2B); and Ragweed (11.8%), Cottonwood (11.1%), Mugwort (Art v1) and Russian thistle (Sal k) (both 7.6%) weeds (Supplementary Figure 2C). Clinical white bean allergy is very uncommon and thus this likely represents cross-sensitisation. The commonest PR-10 and Profilin sensitisations were strawberry (Fra 1 + 3) and melon (Cuc m 2) in 5.8%, followed by hazelnut (Cor a 1.0401) and apple (Mal d 1) with 3.6%, peanut (Ara h 8) in 2.9%, soy (Gly m 4) in 2.3%, carrot (Dau c 1) in 1.7% and 1.5% in celery (Api g 1) (Figure 2A). In individuals sensitized to hazelnut, apple, soy and/or peanut, Rye (62.8%; Lol p 1), Bermuda (Cyn d) and Timothy grasses pollens (Phl p 1) (both 55.8%) sensitisation was common, followed by Hazel (55.8%; Cor a 1.0103), Silver birch (51.2%; Bet v 1) and Beech (48.8%; Fag s 1) tree pollens. In individuals sensitized to celery or carrot, grass pollens sensitisation rates were highest including Rye (85.7%); Bermuda and Timothy grasses pollens both 71.48% (Figure 2C), followed by tree pollens sensitization patterns of Silver birch (57.1%) followed by Alder (Aln g 1), Hazel, Cypress (Cup a 1) and Walnut (Jug r pollen) pollens all being present in 50.0%. None in these two groups were sensitized to any weed pollen. In contrast, individuals sensitized to melon showed substantial grass sensitisations including Bermuda (84.2%), Rye (81.6%) and Timothy grasses pollens (78.9%) (Figure 2D), high tree pollens sensitization to Date palm (94.7%; Pho d 2), Silver birch (71.1%; Bet v 2) and Walnut pollen (60.5%), and weed pollens such as Cottonwood (18.4%; Pop n) and Ragweed (13.2%; Amb a 1).

**Table 3:** Common food allergen-components sensitisation.

Allergen source (organism)	Allergen abbreviation and component	Number of participants sensitised (*n* = 708)	Percentage (%)
White bean	Pha v	168	23.7
Peanut	Ara h 1 (7/8S Globulin)	58	8.2
	Ara h 9 (nsLTP)	44	6.2
	Ara h 6 (2S Albumin)	42	5.9
	Ara h 2 (2S Albumin)	41	5.8
	Ara h 3 (11S Globulin)	40	5.6
	Ara h	24	3.4
	Ara h 8 (PR-10)	22	3.1
	Ara h 15 (Oleosin)	9	1.3
Oyster	Ost e	57	8.1
Apple	Mal d 3 (nsLTP)	45	6.4
	Mal d 1 (PR-10)	25	3.5
	Mal d	17	2.4
	Mal d 2 (TLP)	7	1.0
Hazelnut	Cor a 8 (nsLTP)	33	4.7
	Cor a 11 (7/8S Globulin)	33	4.7
	Cor a 1.0401 (PR-10)	25	3.5
	Cor a 9 (11S Globulin)	22	3.1
	Cor a 14 (2S Albumin)	15	2.1
	Cor a 12_RUO (17 kDa Oleosin)	4	0.6
	Cor a_hazel	4	0.6
Black tiger shrimp	Pen m 1 (Tropomyosin)	32	4.5
	Pen m 2 (Arginine kinase)	29	4.1
	Pen m 3 (Myosin, light chain)	6	0.9
	Pen m 4 (Sarcoplasmic calcium binding protein)	6	0.9
Egg	Gal d_white	27	3.8
	Gal d 1 (Ovomucoid)	25	3.5
	Gal d 5 (Serum albumin)	17	2.4
	Gal d_yolk	16	2.3
	Gal d 2 (Ovalbumin)	15	2.1
	Gal d 3 (Ovotransferrin)	14	2.0
	Gal d 4 (Lysozyme C)	14	2.0
Walnut	Jug r 2 (7/8S Albumin)	21	3.0
	Jug r 1 (2S Albumin)	19	2.7
	Jug r 6 (7/8S Globulin)	15	2.1
	Jug r 3 (nsLTP)	14	2.0
	Jug r 4 (11S Globulin)	14	2.0
	Jug r_nut	1	0.1
Cow’s milk	Bos d 8 (Casein)	20	2.8
	Bos d_milk	15	2.1
	Bos d 4 (*α*-Lactalbumin)	14	2.0
	Bos d 5 (*β*-Lactoglobulin)	11	1.6
Wheat	Tri a 19 (Omega-5-gliadin)	11	1.6
	Tri aA/TI (Alpha-amylase trypsin inhibitor)	7	1.0
	Tri a Gliadin	6	0.8
	Tri a	5	0.7

### 3.4 Fluctuation of grass pollen specific IgE by time of year

Supplementary Figure 3A–C examines average sIgE levels for difference grass species, as well as the number of patients presenting per month to detect alterations in IgE levels or consultation numbers given the seasonality of allergens. Two peaks average sIgE levels across Timothy, Rye (Figure 2C) and Bermuda |(Figure 2B) grass seasons were noted: one matched to the typical grass season in September/October (2) and another during April/May. Similar, although less pronounced, peaks in numbers of consultations with grass sensitisation was noted for September/October and April/May (Figures 2B,C).

## 4 Discussion

Understanding sensitisation patterns can inform strategies for more parsimonious or panel testing, help guide allergen immunotherapy decisions and/or understand AIT non-responders, and is increasingly important to provide baseline data against which to track the impact of our changing environment on allergic sensitisation (13). Multicomponent array testing also allows insights into cross-sensitivity patterns, and to our knowledge this is the first data from the region using the ALEX® and ALEX^2^® multiplex assay that differs from the ISAC primarily by having inhibitors that block the binding of IgE to cross-reacting carbohydrate determinants (CCDs) (14). Our study confirms known patterns of aeroallergen sensitisation for a coastal temperate region, but comparative data demonstrates increasing pollen sensitisation, with the inadequacy of limited panel testing for certain pollen allergen groups such as trees evident. Our data highlights the utility of CRD to inform AIT decisions and likely outcomes given the importance of dual C3 and C4-grass sensitisations (C3 (cool-season) and C4 (warm-season) grass pollens) and the significant number of patients having only minor allergen sensitisations to *B. tropicalis*, dog and cat—the majority of which are found at low concentrations in available immunotherapy products. In contrast to mediterranean regions, melon profilin sensitisation was commonest reflecting predominant primary grass pollen sensitisation with low rates of birch pollen sensitisation consistent with aerobiological monitoring data (2). Finally, although no data is available for food allergy, consistent with existing multiplex data a significant amount of likely clinical irrelevant cross-sensitisation between pollen and food allergens is detectable from these tests e.g., white bean, and this highlights the need for clinicians to always interpret multiplex results together with a clinical history and the potential dangers of first-line multiplex testing in primary care allergy practices or direct-to-consumers.

HDM sensitisation was the leading allergen in this coastal cohort, consistent with previous SPT data from the 1990s (7) and more recent sIgE data (17). Most patients showing dual sensitisation to Der p and Der f allergens, with more than half co-sensitised to *B. tropicalis*. No monosensitisations to Der p, Der f or *B. tropicalis* were found. Van Rooyen et al. showed a similar pattern with SPT data from the Western Cape (17). There is considerably climatic variation of temperate, humidity and altitude across the biomes of SA known to influence HDM in dust samples, with lower levels noted from inland regions and increasing *B. tropicalis* sensitisation in higher latitude coastal regions with increasing humidity and temperature (18). Aligned with this is the lower HDM sensitisation rates reported by Van Rooyen et al. for sIgE and Murray et al. with the ISAC multiplex assay from inland regions (17, 19). Der p 23 was the leading HDM component, with sensitisation in 80% of patients sensitised to Der p; this is a rate higher than in other studies and may be clinically important given known association with severe asthma (20). We also noted 41.7% of patients sensitised to *B. tropicalis* reacted to only a minor allergen (Blo t, Blo t10, Blo t 21). We predominantly use HDM-immunotherapy products that include *B. tropicalis*, with uncertain concentrations of minor allergens, and our clinical experience is that this may be responsible for AIT treatment failure. We therefore recommend the use of component testing to guide shared decision making for HDM immunotherapy in SA.

Pollen allergy is predicted to increase several fold in coming decades as a result of anthropogenic climate change (21), although there is limited data from the Southern Hemisphere (22). Interestingly, although increases in the annual pollen indices for grasses have not been as pronounced, Australian data has indicated a shift in the ratio of C4 to C3 grasses particular in temperate climates (23). Available SPT data from Cape Town in the early 1990s shows sensitisation rates for SA grasses around 30%, with Bermuda—a C4 grass—sensitisation in <10%. In contrast, our data shows a seroprevalence of 45% for any grass pollen with more than three quarters sensitised to Bermuda, demonstrating both the growth of grass pollen sensitisation as well as supporting the growing importance of C4 grasses in our setting. Our data is consistent with recent data from SA (17, 19). Sensitisation rates to any tree pollen was also noted in a third of patients, increasing compared to historical data. The current panel testing for tree pollens by the allergic rhinitis working group (16) recommends initial tree screening include Plane and Cypress tree pollens based on aerobiological data from the SA pollen monitoring network (2). This data supports testing for plane tree as the leading single tree allergen, but highlights the wide diversity in tree pollen sensitisation with 14.4% of patients sensitised to a tree other than Plane or Cypress. This is consistent with data from other parts of SA which shows a wide diversity in tree pollen sensitisation patterns (24). Similarly, current panel testing does not include any weed pollen testing, yet this data indicates sensitisation in nearly one third of patients. Clinicians should consider tree and weed pollens as important triggers in patients with uncontrolled seasonal symptoms not detected on initial panel testing, and alert patients to tree and weed pollens seasons (https://www.pollencount.co.za) and local neighbourhood exposures given the majority of tree allergies in SA are from ornamental, imported tree species that are planted in urban environments and hence show substantial small-area variation.

Multiplex allergen assays are useful tools to understand locally relevant pollen-food allergy syndromes (PFAS) caused by cross-reaction of a specific pollen antigen with a corresponding food allergen in sensitised individuals. PFAS show substantial geographic variations based on available pollen and there is little data from the Southern hemisphere (25). The highest rates of potential cross-reacting pollen-food allergen groups were for the melon profilin (Cucurbetaceae) and strawberry PR-10 and LTP (Roseaceae), with lower rates of apple (Rosaceae) and celery/carrot (Apiace) sensitisation (Figure 2). Primary sensitisation between Timothy grass seems responsible for Cucurbitaceae (melon) cross-sensitisation, while patients with carrot or celery PR-10 likely arose from both Timothy and mugwort primary sensitisation. Birch-pollen levels are low in the Cape and this is reflected in the corresponding lower rates of Rosaceae and Hazelnut sensitisation.

Our data, together with recent sIgE, multiplex ISAC and SPT data, highlights the growing burden of sensitisation to furry animals, particularly cat and dog. This pattern has been noted globally with homes frequently containing dog and cat allergens even when there are no pets in the home (11), and amongst adults prevalence of sensitization to cat and dog allergens was noted to increase during the COVID-19 pandemic (26). The high rates of minor allergen sensitisation to particular dog allergens has been noted as a complexity for successful AIT to animals, and our data is consistent with these international cohorts (27). Novel strategies, such as anti-Fel d 1 immunoglobulin Y in cat food are exciting and would be applicable to our population with only 10% of cat sensitised patients without Fel d 1 sensitisation (28).

Our study is limited by its retrospective data and the lack of individual level clinical allergy data. Consequently, we are not able to draw robust conclusions particularly around food allergy vs. sensitisation. However, we are aware of very few patients with clinically significant allergy to white bean despite one fifth of this cohort showing sensitisation. This highlights the absolute requirement for clinicians to interpreting multiplex IgE sensitisation together with clinical history to avoid unnecessary dietary exclusions and potential harms (29). Related to this, the high rates of CCD MUXF3 positivity (20%) reported by Murray et al. using the ISAC in another South African cohort supports the utility of specific CCD inhibitor in the ALEX^2^® multiplex format; sensitisation rates of just 3% to walnut Jug r 2 which contains CCD illustrating the effectiveness of ALEX^2^® CCD inhibition (19).

In conclusion, this study from a cohort of patients seeking allergy specialist care in Cape Town, South Africa, provides valuable data on primary and cross-reactive sensitisation patterns using the multiplex IgE ALEX® and ALEX^2^® assays. This data provides valuable information for practicing clinicians to help guide testing strategies and the use of allergen immunotherapy. In addition, particularly for pollen and fungal sensitisation patterns, this data provides a valuable baseline from which climate-driven changes can be tracked.

## Data Availability

The original contributions presented in the study are included in the article/Supplementary Material, further inquiries can be directed to the corresponding author.
