# Supplementary material for: Analysis of the multicomponent ALEX array data to examine patterns of sensitization in Cape Town, South Africa
Source: Front Allergy. 2025 May 7;6:1572509. doi: 10.3389/falgy.2025.1572509 (PMC12094254; doi:10.3389/falgy.2025.1572509)
Supplement: Supplementary file 1 [file Datasheet1.pdf]

Supplementary materials

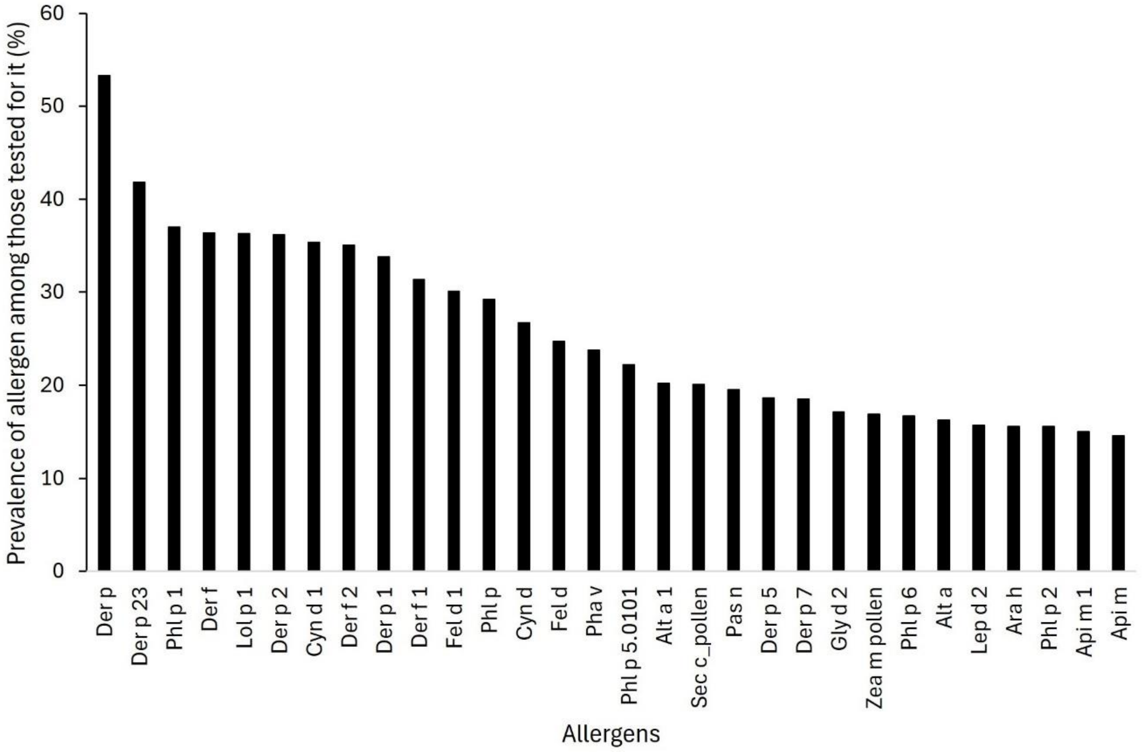

Supplementary Figure 1: Percentage prevalence of 30 most commonly sensitising allergens in the studied population (n=708).

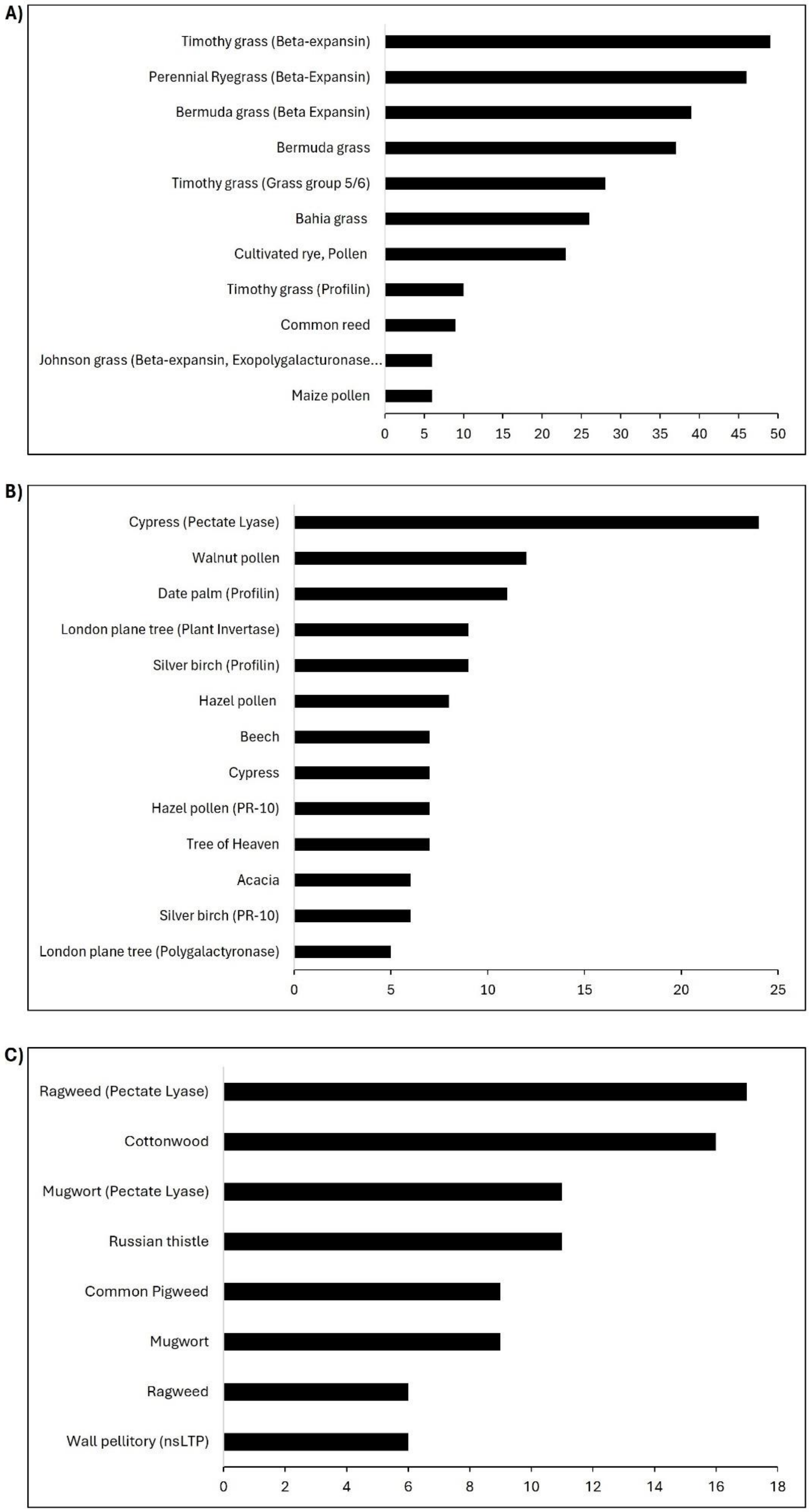

Supplementary Figure 2. A) Grass B) tree and C) weed pollens patterns in adult patients sensitized to white bean (n=144).

Commonly cross-reactive compound	n^2	n^1	'Degree' of cross-reactivity (n^2/n^1 %)
**NPC2**	251	267	94.0
**Profilin**	50	63	79.4
**Tropomyosin**	32	48	66.7
**nsLTP**	68	108	63.0
**PR-10**	35	58	60.3
**Lipocalin**	78	148	52.7
**Uteroglobin**	0	213	0.0

Supplementary Table 1: Commonly cross-reactive compounds and the proportion of sensitised participants that are reactive to the compound across at least two distinct allergen groups.

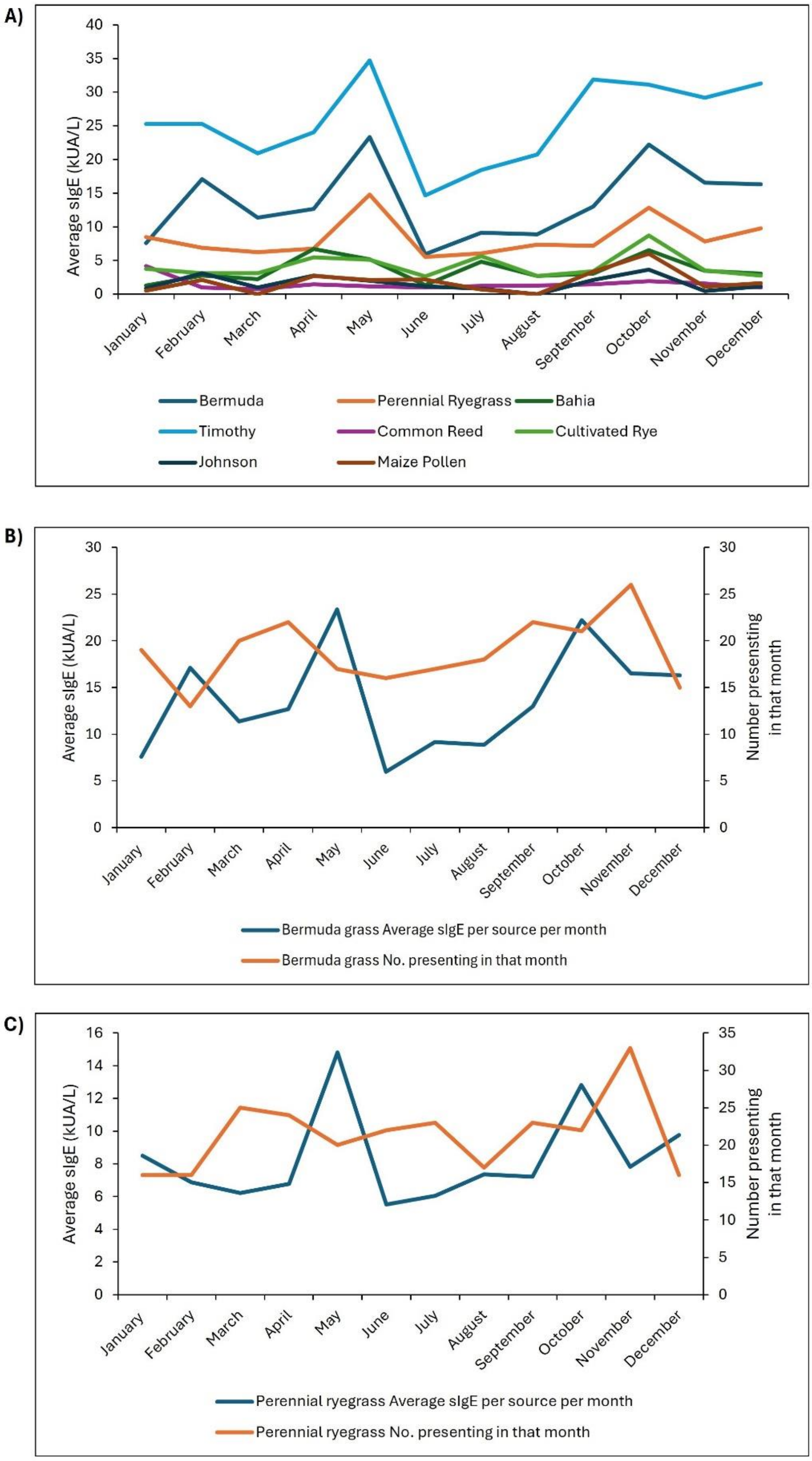

Supplementary Figure 3. A) Summary of seasonal grass pollen sIgE levels in those sensitised to grass pollens (n=319), B) Bermuda grass seasonality pattern by average sIgE among those sensitised to Bermuda grass (n=189) and total number of patients presenting in that month (2019 – 2024), C) Perennial ryegrass seasonality pattern by Average sIgE among those sensitised to Perennial ryegrass (n= 57) and total number of patients presenting in that month (2019 – 2024).

Overarching category	Allergen group	Allergen sources included per group
**Mites and cockroaches**	House dust mites	American house dust mite, Blomia tropicalis, European house dust mite
	Cockroaches	American cockroach, German cockroach
	Storage mites	Acarus siro, Glycyphagus domesticus, Lepidoglyphus destructor, Tyrophagus putrescentiae
**Pollens**	Tree pollens	Acacia, Alder, Ash, Beech, Cottonwood, Cypress, Date palm, Elm, Hazel, Lilac (ALEX 1), London plane tree, Mountain cedar, Mulberry, Oak (ALEX 1), Olive, Paper mulberry (ALEX 2), Privet (ALEX 1), Silver birch, Sugi, Walnut
	Grass pollens	Bahia grass, Bermuda grass, Common Reed, Cultivated rye, Johnson grass (ALEX 1), Maize (ALEX 1), Perennial ryegrass, Timothy grass
	Weed pollens	Annual mercury, Common pigweed, Hemp (ALEX 2), Lamb's quarter, Mugwort, Nettle, Ragweed, Ribwort, Russian thistle, Sheep sorrel (ALEX 1), Wall pellitory
**Dander and epithelia**	Pets	Cat, Dog, Guinea pig, Hamster, House mouse, Rabbit, Rat
	Farm animals	Cattle, Goat (epithelium), Horse (epithelium), Pig, Sheep (epithelium)
**Moulds and yeasts**	Moulds	Alternaria alternata, Aspergillus fumigatus, Cladosporium herbarum, Penicillium chrysogenum
	Yeasts	Candida (ALEX 1), Malassezia sympodialis, yeast
**Foods**	Meats	Cattle, Chicken, Horse, House cricket (ALEX 2), Mealworm (ALEX 2), Migratory locust (ALEX 2),Pork, Rabbit, Sheep, Turkey
	Fruits	Apple, Banana, Blueberry, Cherry, Fig, Grape, Kiwi, Litchi (ALEX 1), Mango, Melon, Orange, Papaya, Peach, Pear, Plum (ALEX 1), Raspberry (ALEX 1), Strawberry
	Vegetables and mushrooms	Avocado, Cabbage (ALEX 1), Carrot, Celery, Garlic, Lettuce (ALEX 1), Olive (ALEX 1), Onion, Potato, Tomato, White mushroom (ALEX 1)
	Legumes	Chickpea, Lentil, Pea, Soy, White Bean
	Seafood	Atlantic cod, Atlantic mackerel (ALEX 2), Black tiger shrimp, brown shrimp (ALEX 2), Carp, Clam, Common mussel, Crab, Herring worm, Lobster, Oyster, Salmon, Scallop, Shrimp, Squid, Thornback ray (ALEX 2), Tuna
	Nuts	Almond, Brazil nut, Cashew, Hazelnut, Macadamia, Peanut, Pecan, Pistachio, Walnut
	Cereals	Barley, Common buckwheat, Cultivated rye, Lupine seed, Maize, Millet, Oat, Quinoa, Rice, Spelt, Wheat
	Milk	Camel, Cow, Goat, Mare's, Sheep
	Spices	Anise, Caraway, Mustard , Oregano, Paprika, Parsley
	Seeds	Fenugreek (ALEX 2), Poppy, Pumpkin, Sesame, Sunflower
	Egg	Egg white, Egg yolk
**Hymenoptera venoms**	Honey-bee venoms	Honey-bee
	Wasp venoms	Hornet, Paper wasp, Wasp
	Fire ant poison	Fire ant (ALEX 2)
**Other**	Ficus	Weeping fig
	Latex	Latex
	CCD (cross-reactive carbohydrate determinants)	Ananas (ALEX 1), Hom's lactoferrin
	Parasite	Pigeon tick (ALEX 2)

Supplementary Table 2: Details of the allergens in each group used for table 1.
